# Word meaning types acquired before vs. after age 5: implications for education

**DOI:** 10.3389/fpsyg.2024.1280568

**Published:** 2024-04-05

**Authors:** Andrew Biemiller

**Affiliations:** Dr. Eric Jackman Institute of Child Study, Ontario Institute for Studies in Education, University of Toronto, Toronto, ON, Canada

**Keywords:** children’s vocabulary, concrete, nonverbal, symbolic, abstract, verbally-defined

## Abstract

This article concerns two types of word meanings: nonverbal meanings which appear to be associated with neurological representations and verbally-based meanings which appear to depend in part on other words to construct meanings. Using word use data from Hart and Risley’s study of children aged 19 to 36 months, and word meaning knowledge data from Biemiller and Slonim’s studies of children between aged 5 to 11, meanings were classified as nonverbal or verbally-based. Biemiller and Slonim used sampled word meanings reported known from grade levels 2 to 12 reported by Dale and O’Rourke in their Living Word Vocabulary. Virtually all meanings used at age 3 or known at age 5 (preschool) were classified nonverbal. By grade two, and even more by grade five, children had added many verbally-defined meanings, although by grade five the majority of the word meanings known were still nonverbal. Evidence for neurological meaning associates are cited. Implications for vocabulary support and instruction at various ages suggest that for children under 6, supporting larger nonverbal vocabularies while after age 6 should prioritize verbally-defined meanings.

## Introduction

In this article, I will be discussing how types of word meanings change with age, and their implications for instruction. Lawson-Adams and Dickinson (2020) note that meaning types form a dimension of “representational modalities” including nonverbal meanings and verbally-based meanings.^1^ No doubt, more distinctions can be made, particularly as children expand their vocabularies after first or second grade.

The distinction between nonverbal vs. verbally-defined meanings is based on the hypothesis that nonverbal meanings are associations between oral wordforms and neurological representations of perceived phenomena (mainly in the posterior cortex) and procedural organized action (mainly in the anterior cortex) plus some modifiers and functors. Verbally-defined meanings are at least in part constructed out of other meanings (Deacon, 1997; Nelson, 2007). In my previous studies of vocabulary development (Biemiller and Slonim, 2001; Biemiller, 2005, 2010a,b), I was not alert to the word type distinction between nonverbal vs. verbally-defined word meanings. The previous work showed that word meanings are acquired in a predictable order and that “priority” words could be identified using this sequence. I now think that the distinction between nonverbal and verbally-defined meanings is important for educators interested in fostering vocabulary development in schools. I will be presenting data from 3 sources that show that before age 5, most or all acquire word meanings have nonverbal neurological representations. These provide the basis for constructing verbally-based meanings after age 5. However, the data presented here indicates that children in the primary grades (K—2) continue to acquire more nonverbal than verbally-based meanings. Those with larger vocabularies can acquire more verbally-based meanings. Most of what are considered “academic vocabulary” are verbally-based meanings. I will argue that building adequate vocabularies will involve attention to acquiring enough nonverbal word meanings prior to age five, and continuing addition of nonverbal meanings but with much emphasis on verbally-defined word meanings in the primary grades.

The practical significance of different word meaning types is relevant for encouraging larger vocabularies, especially for children who acquire significantly below-average size vocabularies. There is clear evidence that children with smaller vocabularies as early as age 5 or younger tend to comprehend read texts less well in later years.

Vocabulary accumulation begins early. The size (breadth) of children’s vocabulary knowledge at kindergarten predicts their reading skills (including reading comprehension^2^) through fourth grade (Scarborough, 2001; Dickinson and Porche, 2011). Those predictions are based on the size of PPVT vocabulary assessments in kindergarten (picture-based), which are limited mainly to “concrete” meanings (what I refer to as “nonverbal meanings”). Cunningham and Stanovich (1997) showed that vocabulary size (PPVT) in grade one was predictive of reading comprehension as late as grade 11. In addition, Hart and Risley (1995) reported that the number of words *used* by children by age 3 was predictive of vocabulary in grade three, using both the PPVT-R and TOLD vocabulary tests with 29 of their original 42 children.

### Neurological basis of nonverbal meanings

These meaning representations are long-evolved neurological organizations in animals. Neurologically there are six levels of perceptual and procedural processes in the human cortex (Luria, 1973; Pinel and Edwards, 2008). Vertebrates—including humans—acquire *perceptual* information from senses about *objects* and *agents* (spontaneously moving objects), *settings* (places where objects, events, and actions occur), *events* (sequences of actions on objects), *sounds*, etc. (Gibson, 1969, 1979). If the Gibsons are correct, perceptual processes create neurological representations for objects, settings, and events, among other sense organ inputs. Without such representations, animals could not distinguish between “familiar” vs. “novel” objects, sound patterns, etc. These neurological “representations” are similar to digital “files” in computers. They can be stored, retrieved, and used in perception. Similarly, vertebrates acquire and inherit *procedural* information about actions and organized procedures. Procedural processes also create neurological representations (neurological files) that can be stored and accessed for complex instinctive actions. Lorenz (1981)describes details of instincts (Lorenz, “fixed action plans” or FAPs). For both neurological representations and digitally-organized information, “file” is a metaphorical term, an analog of a physical “file” in which we store written or graphic information that can be retrieved.

The perceptual components are mainly located in the posterior (rear) cortex, while the procedural components are largely located in the anterior (front) cortex of the brain. Luria (1973) has described layers of brain functions. The perceptual areas have inner layers where raw input from sense organs arrive (afferent neurons) to at outer levels where raw inputs are organized into perceptions of objects, settings, identifiable sounds, etc. The procedural areas where output impulses for actions at the lowest cortex level (efferent neurons) are directly connected with muscles to activate them. In the outer procedural layers, procedures are organized to produce goal-oriented actions to capture prey, find edible food, care for young, mate, etc. Luria’s book was written 50 years ago. However, much more recent neurological maps appear to be in agreement with Luria’s model (e.g., Pinel and Edwards, 2008, ch. 7). In addition, many *tasks* must include connections between procedures and perceptions (e.g., hunting a mouse.). Neurological programs integrating raw information and movement into ways of surviving and reproducing probably includes some “behavioral grammar,” as suggested by Lashley (1951).

### Nonverbal meanings

Paivio (1986), Clark and Paivio (1991), and Lawson-Adams and Dickinson (2020) argue for *two* representational types for word meanings: nonverbal and verbally-based. I differ from them (and from Deacon, 1997; Nelson, 2007) in hypothesizing that most children *below* ages 4.5–5.5 associate *most* spoken word files (“wordforms”) with nonverbal meanings (neurological organizations or “mental representations” or files), including:

- *Perceptual* (nouns—objects, settings, events).^3^ what we classify as “nonverbal” *nouns* are either physically perceived objects (including agents), settings, or events, or other perceptually established neurological files (representations) from senses. These perceptual processes are mainly located in the posterior cortex.
- *Procedural* (verbs—actions, tasks). What we classify as “nonverbal” *verbs* are action components (e.g., *grasp*), or more complex actions (e.g., *pounce*) or task programs (e.g., beaver constructing a lodge) procedurally established as neurological files (representations). These procedural processes are mainly located in the anterior cortex but must have connections with the perceptual processes in the posterior cortex.
- *Modificational* (adjectives or adverbs—discriminating between similar perceptions or procedures). What we classify as nonverbal *adjectives* or *adverbs* refer to specific features of perceptions (e.g., *big* vs. *small* objects) or features of procedures (e.g., *quickly* vs. *slowly*).^4^ I do not know where modificational neurological files are likely to be located in the brain.
- *Functional* (functors—prepositions, conjunctions, articles, demonstratives, pronouns, and probably interrogatives). Words generally activate parts of both the posterior, temporal, and anterior cortices. Function words differed by *less* activation in the anterior cortex.

### Neurological evidence for nonverbal meanings

Tomasello et al. (2017) report evidence of neurological activity for “Brain connections of words, perceptions and actions.” R. Tomasello’s evidence for neurological organization of nonverbal word meanings:

… some additional cortical areas contribute to semantic processing in a more selective fashion, being particularly relevant for specific semantic categories, such as words typically used to speak about animals, tools, or actions and their related concepts. Some evidence also indicates that when recognizing a word such as run, activity in motor cortex, and even more specifically in leg-motor cortex, emerges, whereas, when hearing an object-and visually-related word such as sun, activity in visual areas is relatively more pronounced (Damasio et al., 1996; Hauk et al., 2004; Boulenger et al., 2009; Pulvermüller et al., 2009; Gainotti, 2010).

In other words, many word meanings involve neurological centers for procedural processes (action-meanings) or perceptual processes (sense-related meanings).

Diaz and McCarthy (2009) studied neurological activations to “content” words (nouns, verbs, and adjectives) vs. “function” words.

Both word types strongly activated temporal-parietal posterior cortex, middle and anterior temporal cortex, inferior frontal gyrus (lobe or fold in cortex AB), parahippocampal gyrus (cortex associated with memory, AB), and orbital frontal cortex. Activations were more extensive in the left hemisphere. Content words elicited greater activation than function words in middle and anterior temporal cortex, a sub-region of orbital frontal cortex, and the parahippocampal region. Words also evoked extensive deactivation, most notably in brain regions previously associated with working memory and attention.

In other words, the neurological correlates of words activated parts of the posterior and temporal cortices and the front of anterior cortex. “Content” words differed from function words by greater activation in various parts of the temporal and anterior cortex regions.

### Verbally-based meanings

Around age 4.5 to 5.0 or later, children become able to acquire *some* verbally-based word meanings. As discussed by Deacon (1997) and Nelson (2007), verbally-based word meanings are constructed in part from other, often nonverbal word meanings. Deacon and Nelson refer to these as “symbolic” words. For example, the meaning of *height* involves understanding a dimension that ranges from *short* to *tall.* Following Case (1985), acquiring these verbally-based meanings involve increased working memory around age 5 making possible for children to attend to relations between nonverbal meanings such as *short* and *tall*. These also appear to be the same as Lawson-Adams and Dickinson’s (2020) “lexical” meanings (also what I consider to be verbally-based meanings). These verbally-based “word meanings” go beyond nonverbal *perceptual, procedural*, *modificational,* and *functional/grammatical* meanings. Other examples include, simple verbally-based “dimensional” words such as *weight* (vs. *light* and *heavy*^5^), or *social class* (vs. *rich* and *poor*).

Reggin et al. (2021) discuss variables affecting acquisition of abstract words. They summarize: “Thus, (emotional) valence, interoceptive strength, and mouth action strength facilitate acquisition of abstract words, and are less important for acquisition of concrete words, consistent with the predictions of the embodiment account. These results provide further evidence to support the claim that abstract words are grounded in emotional and associated sensorimotor experiences.” The role of linguistic experience—range of other words encountered with a target word—was also a factor. Reggin et al. state, “the current findings suggest that all words, both concrete and abstract, are learned earlier when they have been experienced in diverse contexts, presumably because their meanings have greater opportunities to be linked to already known words.”

Many basic “dimensional” words such as *height, weight, length,* and *size*, are considered “known” by grade four in the readability scale of Chall and Dale (1996). [However, many of these meanings are really only learned by 67%–80% of grade four children in their samples as reported in the *Living Word Vocabulary* of Dale and O’Rourke (1981).^6^]

Other examples of words with verbally-based meanings are: *peace, astronomy, democracy,* or *liberty.* These more “abstract” meanings cannot be referred to simple things we perceive (perceptual files), or simple actions or combinations of actions into tasks that we carry out (procedural files). Note that these verbally-based meanings are very rarely assessed in picture vocabulary tests. None of these verbally-based words appear in Hart and Risley’s (1999) sample of words used by age 3.

In addition, after age 5, children’s ability to comprehend and produce longer narratives increases substantially. Case and his colleagues have documented growth in working memory correlated with cognitive changes (including length of sentences and narratives) around these ages as have others (Case, 1985; Case and Okamoto, 1996; Gathercole et al., 1997, 1999).

The grammatical classification of verbally-based meanings include nouns, verbs, modifiers (adjectives and adverbs), and functors. Note that this classification parallels the functional classifications based on neurological functions that I have used to describe nonverbal meanings. These grammatical classes of words continue to be necessary to construct meaningful sentences as children develop.

One theory of word meanings is that verbally-based meanings are constructed from co-occurrences of a specific words and its environment of other words in paragraphs in various texts (Landauer, 2007; Biemiller et al., 2014). Landauer and associates refer to these meanings as “Latent Semantic Meanings” (Landauer, 2007). Note that after age 5, children can supply verbal meanings for nonverbal meanings as well as for verbally-defined meanings. Landauer (2007, p 23) suggests that latent semantic meanings are acquired “…not all or none, but grows gradually, not showing itself outwardly until good enough and in the right context with the right measuring instrument.” However, Carey (1978) notes that some words can be “fast-mapped”—acquired from one or two experiences. I suggest that “fast-mapping” may be limited to nonverbal meanings.

Hadley and Mendez (2021) give a good illustration of the difference in meaning types between nonverbal meanings and symbolic or lexical meanings:

Consider the difference between learning a word like “gunwale” vs. a word like “analyze.” For “gunwale,” prior knowledge or morphological strategies would be of little help, but a quick reference to a picture would reveal the word’s meaning (the upper edge of a boat). However, one would rarely, if ever, encounter the word “gunwale” again, and might quickly forget it. To learn the word “analyze,” a picture or even a definition might not be helpful, but hearing multiple examples and uses in context could build a working knowledge of the word (Bolger et al., 2008). Since “analyze” is used frequently across domains, a child’s knowledge of this word would continue to grow as they encountered it over time. Moreover, learning this word gives a child a valuable tool in making meaning from text.

Hadley et al. (2021) have studied concrete vs. abstract words included in a vocabulary intervention study with preschool children (average age 4.9 years). I believe these are similar to the distinction I draw between nonverbal meanings (concrete) and verbally-based meanings (abstract). In Hadley et al.’s article, they instructed 20 words that were above the “easy” level reported by Biemiller (2010a,b). Their results show that four of the five the most learned words were “nonverbal meanings” (could be files of perceived or procedural neurological stored information, e.g., *liquid, sway*). Their average score was 0.86 out of 6 possible. A score of 1 refers to one type of correct response (e.g., synonym or perceptual feature). Among the other 15 words are a mix of 6 non-verbal and 9 verbally-based meanings. The nonverbal meanings are rarely-encountered words in preschool (e.g., *glade, struggle*). Most of these words were not well learned. The verbally-based meanings were also rarely encountered meanings (e.g., *strategy, meander*). The average score for these 15 instructed words was 0.34 out of 6 possible. (The test words and data have been supplied by E. Hadley, personal communication.) It appears that teaching rare words to preschoolers was not very useful.

### More fine-grained verbally-defined word meanings

Uccelli et al. (2014) provide a more fine-grained description of advanced verbally-defined meanings—that they describe as Core Academic Language. “In contrast, CALS refers to language skills that cut across content areas and are used to fulfill similar language goals, such as communicating or understanding precise meanings, concisely packed information, and explicitly marked conceptual relations.” Their work makes clear that this more advanced language continues to develop between fourth and eighth grades and is a dimension rather than a binary distinction between nonverbal vs. verbally-defined meaning types.

### Neurological evidence for verbally-based meanings

Papagno (2022) have summarized research on neurological studies contrasting “concrete” vs. “abstract” word meanings. Their summary states,

The anatomical implication is that abstract concepts are represented entirely verbally, in the left hemisphere, whereas the representations of concrete concepts have both left hemisphere, verbal components, and right hemisphere, visuo-perceptual components.” Specifically, activation of neurological activity with abstract (verbally-defined) words occur in the left middle longitudinal part of the temporal lobe (middle temporal gyrus AB) and the lower left bottom part of the anterior cortex (left inferior frontal gyrus). These brain areas relate to perceiving and producing words. Concrete (nonverbal) meanings activate these left brain areas more strongly, and also activate parts of the posterior cortex on both sides of the brain.

### Becoming able to explain (define) word meanings

At about the same age (5 years) when children begin to acquire *verbally-based* meanings, children also become increasingly able to supply verbal “meanings” for words—especially when the requested word has been “illustrated” with a context sentence. Prior to age 5, children are very limited in verbally explaining word meanings, although they can pass “picture vocabulary” test items and follow verbal instructions—when they “know” the words in an instruction (Meichenbaum and Biemiller, 1998; Biemiller and Slonim, 2001). By 2012, Wechsler (2012) and others no longer requested word meanings from children under 6 as part of language or intelligence assessments. This reflects the fact that children under 5 often cannot supply a word meaning despite “comprehending” words.

Once children become able to create and understand verbally-defined word meanings, they may create verbal meanings for what have been nonverbal meanings.

However, our data (Biemiller and Slonim, 2001) indicates that before the end of grade two, children were limited in supplying word meanings. They appeared to acquire a great many word meanings during grade two in Biemiller and Slonim’s cross-sectional data. Biemiller and Slonim have suggested that this reflected a limitation in acquiring verbal meanings before age 7 or 8. Cross-sectional data in Biemiller and Slonim (p 509) suggested that an estimated average of about 3,000 root word meanings were added around grade two, whereas for other grades, an estimated average of about 1,000 root word meanings were acquired. This “leap forward” in grade two probably reflects (a) increased formation of “latent semantic meanings” extracted from repeated receptive uses of a word form in various oral and written contexts, plus (b) increased ability to verbally describe information in nonverbal perceptual or procedural representations. [However, when verbal definitions are *taught,* they can be learned before age 5 (Hadley et al., 2016).]

I expect that after age 5, once children become able to create and understand verbally-defined word meanings, they may add verbal meanings for what have been nonverbal meanings. This can occur either by extracting meaning from verbal contexts or by describing information stored in visual perceptual files. They are also acquired when explained verbally by another person (Biemiller, 2005). In addition, more than one meaning can be associated with a word-form—both nonverbal and verbally-defined meaning types (e.g., *strike—*to hit; *strike—*a discovery).

## Methods

### Research questions

1. Study 1. Are most or all of word meanings known at age 3 nonverbal?
2. Study 2. Do proportions of word meaning types show an increase in verbally-based meanings between age 5 and age 8?
3. Study 3. Do proportions of word meaning types known increase from kindergarten to grade five with attention to growth of verbally-based meanings between grade two (end of primary grades, age 8) and grade five (near end of upper elementary grades, age 11)?
4. Study 3. Are there differences in acquisition of numbers of nonverbal vs. numbers of verbally-defined word meanings for at/below vocabulary children vs. above median children for grades kindergarten to grade five?

### Classifying word meanings as nonverbal or verbally-based meanings

Quite simply, I classify word meanings as *nonverbal* if the meaning *could* be stored mentally as a nonverbal neurological mental file in the perceptual representations (object or setting seen, smelled, tasted, felt, heard, etc.), procedural representations (actions, tasks), nonverbal modificational representations (perceivable or procedural distinctions), or functional representations (relations between objects, settings, actions, time, as well as pronouns and interrogative terms).

I classify word meanings as *verbally-based* if the meaning could not be stored nonverbally without some further verbal qualification. This includes quantitative dimensions, such as *numbers, height,* or *distance*—and qualitative dimensions such as *social class*, or biological *phyla*. Similarly, procedures such as *compute, analyze,* or *perceive* are verbally-based.

Readers may find some word meanings that I have listed in subsequent tables that they would classify differently. I have included all classified words and specific LWV meanings in several appendices in this article [excepting about 90% of the 1,361 words listed by Hart and Risley, 1999, where I assume each word’s most common meaning as reported by Dale and O’Rourke, 1981].

### Three studies to support nonverbal vs. verbally-based word meanings that children know: Study 1—age 3; Study 2—ages 5–8; and Study 3—second and fifth grades and some information re kindergarten to grade five—I also include a vocabulary median split by grade

In this article I will be describing data from 3 studies drawn from a vocabulary book and two published studies concerning children’s word use or knowledge at various ages and adding a classification of meanings acquired as nonverbal vs. verbally-based.

*Study 1:* The purpose of Study 1 was to determine the types of word meanings *used* by children by age 3. These data are from children in Hart and Risley’s (1999) book: *The Social World of Children Learning to Talk.* Children were observed from 19 months to 36 months.

*Study 2*: The purpose of Study 2 was to examine the types of word meanings vocabulary acquired at the end of pre-kindergarten (age 5) to grade two (age 8). These word meanings were sampled from grade levels 2 to 12 in Dale and O’Rourke’s (1981) *Living Word Vocabulary*. These data on knowledge of 59 word meanings were classified as 30 nonverbal meanings and 29 verbally-based meanings in Biemiller and Slonim’s (2001) study. (At the time of the study, we were not attending to non-verbal vs. verbally-defined meanings.) The study population were from advantaged pre-kindergarten to grade two children in a laboratory school. By “advantaged,” I mean a population of college-graduate parents including many professionals, at a university laboratory school.

*Study 3:* The main purpose of Study 3 was to contrast word type acquisition at grades two and five. An additional analysis involved splitting samples in each grade into children at/below vocabulary median vs. above median. These data include knowledge of 117 word meanings known at grade level two to grade 12 for representative English-speaking children in kindergarten through grade five (Biemiller and Slonim, 2001). This study involved a population that included low-income, average, and advantaged elementary students. Again, these word meanings were sampled from Dale and O’Rourke’s *Living Word Vocabulary.*

## Study 1: words used at age 3 (semi-representative sample)

The purpose of Study 1 was to determine the type of word meanings *used* by children by age 3. My expectation was that all or most words used would have *nonverbal* meanings.

Hart and Risley (1999) have a list of 1,361 root words reported used by age 3.0 by 2 or more children in a semi-representative sample of 42 children. (The list of words appeared in their 1999 book.) I analyzed the root words reported *used* by 2 or more children in Hart and Risley’s book. The word meanings were classified as nonverbal vs. verbally-based, and by grammatical function (noun, verb, modifier, or functor) and by how many words were used by their children: most—80% or more; many—79%–40%; or few—39% or fewer. (The “least used” words reported were used by at least 2 children by age 3.)

### Methods: data on words at age 3

#### Sample

This was a semi-representative sample of disadvantaged, working-class, and advantaged children in Kansas. Children were recruited from 3 groups: 13 children from a university laboratory school—parents mostly had college education; 23 children from working class families whose parents mostly had non-college education; and 6 less advantaged children in financially assisted homes (all Black). Study 1’s sample of “disadvantaged” children were recruited from attendees at sessions of the Special Supplemental Nutrition Program for Women, Children, and Infants (U.S. Federal). This yielded the 6 Black welfare-supported families (Hart and Risley, 1995).

The children were roughly “representative” of children in Kansas City, KS around 1990. Their study probably underestimates the proportion of disadvantaged students in the area and ignores the many disadvantaged nonblack population in Kansas City or at large in the U.S.

#### Procedure

Hart and Risley (1999) recorded all child and parent/caretaker language during monthly one-hour home observations. They reported 1,361 root words used by 2 or more children between 19 months and 36 months. Hart and Risley’s Appendix A notes when words were first used by two or more of the children. [These root words are listed by Hart and Risley without reference to grammatical affixes, i.e., inflections. Very few semantic affixes (e.g., re- or -able) appear among these words. (A few irregular past tense forms were used.) (Meaning types classified by the author.)]

### Results: word meanings at age 3

In Study 1, most of the words reported used had clearly *nonverbal* meanings. A substantial number of functors were among the words used early by many or some children.

Table 1 describes most of the 1,361 root words reported used by *most* of Hart and Risley’s sample (80% or higher), *some* (40%–79%), or *few* (39% to 5%—a minimum of 2/42 by age 3 in their 1999 study). Almost all of these words have nonverbal meanings. (There were 28 words that I could not classify.)

**Table 1:** Words used by age 3 by most, some, or few by types and frequency (Study 1).

Meaning type	Words used by			All words
	Most	Some	Few	
	(100%–80% chil.)	(79%–40% chil.)	(39%–5% chil.)	
**Nonverbal**				
Perceptual	32% (60)	54% (145)	58% (492)	53% (697)
Procedural	30% (56)	20% (54)	24% (206)	24% (316)
Modificational	6% (12)	15% (42)	11% (94)	11% (148)
Functional	32% (61)	11% (29)	7% (63)	12% (153)
*Total Nonverbal*	*100% (189)*	*100% (269)*	*100% (855)*	*100%* (1,314)
**Verbally-based** ^**a**^				
Perceptual	(0)	(0)	26% (5)	25% (5)
Procedural	(0)	(0)	(0)	(0)
Modificational	(0)	100% (1)	74% (14)	75% (15)
Functional	(0)	(0)	(0)	(0)
*Total Verbally-based*	*(0)*	*100% (1)*	*100% (19)*	*100% (20)*

Taken from Hart and Risley’s list of words used by age 3 in their 1999 book. Their sample included 42 children. Root words (mono-morphemic) used by 2 or more children are included in these data. ^a^Most possible verbally-based words were numbers over five (e.g., *three, thirty*). I interpret these as modifiers. The remainder were: *year, world, temperature, week,* and *heaven*. (I do not know how these words were understood by these 3 year old children.) ^b^There were 23 words that I could not classify. Some were used by many. Examples are *bye, no, ouch*. Others were used by few. Examples are *welcome, thanks, giddyup*. In addition, the Hart and Risley also includes a number of irregular past tense forms (e.g., *throwed, falled*). I did not count these. Some but not all also appeared in present tense in their Appendix.

These data indicate that about a third of Hart and Risley’s 119 most*-*used words were functors and most of the rest of the most-used words had perceptual or procedural meanings. Among the less well-known words, meanings were about half perceptual, a quarter procedural, and the rest divided between modificational or functional meanings.

Appendix 1 shows a sample of words used by 2 or more of 42 children. In Appendix 1, I have included a sample of 9% of the whole list, yielding a table about the same size as Appendices 4, 5. Among words used by age 3 were *water, milk* (perceptual), *come, say* (procedural), *open, wet* (modificational), and *here, they, with* (functional). These words were used by *most* children (80% or more of the children) by age 3. The words used by *some* (40%–79%) were similar—very “concrete” every-day kinds of meanings. Words used by *few* (39% or fewer) were also very similar. The 4 functors used by few were: *either, or, almost, as.* Readers can see examples in Appendix 1.

Of the 19/1,361 apparently verbally-based meanings, 14 were numbers from 6 to 60. The remaining words were *world, year, heaven, week,* and *temperature.* We cannot tell how well these words were understood without the associated context.

## Study 2: word meanings known at preschool, kindergarten, grade one, and grade two (advantaged sample)

The main purpose of Study 2 was to examine vocabulary acquired by the end of pre-kindergarten (age 5) to the end of grade two (age 8). My hypothesis was that at age 5 there would be few or no verbally-based meanings known. By age 8, I expected that some verbally-based meanings would be known by some or most children. The youngest children were roughly 5 at the time of testing (spring, 1999).

The word meanings tested were sampled from an estimated grade two or lower to grade 12 as determined by Dale and O’Rourke (1981). My samples of the word meanings included 30 meanings that were at least possibly nonverbal and 29 that were verbally-based. Sampling to construct this list of meanings to test was not constrained by nonverbal vs. verbally-based type—that’s simply what they turned out to be. (At the time these word meaning samples were constructed in 1998, we were not aware of their type: nonverbal vs. verbally-based meanings.)

### Methods: data on word meanings known by children from ages 5 to 8

The 59 word meanings used in this study were from Biemiller and Slonim’s (2001) Study 2.

#### Sample

The children in this study attended a university laboratory school. Children were mainly from middle or upper middle-class families. Most parents were college educated. The school did charge tuition. These children could be considered educationally “advantaged.” All children in the study grades were included. This study was conducted in the spring of 1999. The children in the pre-kindergarten group averaged about 5 at the time of testing. Children in the older grades were about 6, 7, or 8 years old. There were 22 children in the pre-kindergarten class, 20 in kindergarten, 22 in grade one, and 22 in grade two.

#### Choosing words

For our analysis of word meaning knowledge among elementary school children, we drew samples of 10 root word meanings from each of grade levels 2, 4, 6, 8, 10, and 12 from Dale and O’Rourke’s, 1981 *Living Word Vocabulary* (LWV; Biemiller and Slonim, 2001). We found roughly 2,500–4,000 *root* word meanings at each LWV grade level (Biemiller, 2005). (One word meaning was dropped from analysis when the test sentence led to 2 different plausible meanings from children.)

#### Procedure

Individual oral testing, words were presented in context sentences, responses written down by examiner. In all test sessions, we would read a test sentence to a child, then ask “What does (*word*) mean?” The child’s response would be written down. For example, “Use a knife to *spread* the jam. What does *spread* mean?”

*Coding responses:* (1—correct—child understands word meaning in sentence to follow a narrative including this sentence; 0.5—not sure whether child knows this meaning; 0.1—wrong response; 0.0—no response. Agreement between raters in a blind test was 90%).

#### Statistical analysis

Analyses of variance and correlations conducted with *Systat 13* (Systat, 2009).

### Results: word meanings by type from ages 5 to 8 (laboratory school sample)

To summarize, before grade one, virtually no *verbally-based* words were known. Only *subtract* was known by some children in kindergarten. By the end of first grade, 4 *verbally-based* word meanings were known by some children, and by grade two, 5 (of 29 tested) *verbally-based* word meanings were known including one known by most (*subtract*). There were significant gains across grades, and significantly more *nonverbal* than *verbally-based* meanings were known. The numbers of nonverbal and verbally-based meanings were substantially correlated for students in each grade.

#### Percent correct

In Table 2, percentages of the 59 word meanings known by meaning type sampled from Dale and O’Roarke’s *Living Word Vocabulary* are shown for pre-kindergarten to grade two. Both types (nonverbal vs. verbally-based meanings) and grades varied significantly at *p* < 0.001 level (Type: *F* = 688.2, 1 and 82 df, repeated measure; Grade: *F* = 36.4, 3 and 82 df). More nonverbal meanings were known, and more meanings were known at older grades. Type and grade interacted significantly, reflecting the increasing percentage of nonverbal word meanings relative to verbally-based meanings in higher grades, as seen in Table 2 (type × grade: *F* = 10.2, 3 and 82 df, also *p* < 0.001).

**Table 2:** Percentages of nonverbal and verbally-based meanings by grade and correlation by type (Study 2).

Grade	*N*	Type of meanings		Correlation nonverbal × verbally-based
		Nonverbal (30 meanings)	Verbally-based (29 meanings)	
Pre-Kdgn.	22	23%	2%	0.73
Kindergarten	20	40%	6%	0.56
Grade one	22	49%	13%	0.70
Grade Two	22	65%	24%	0.75
Total	86	44%	12%	0.80

#### Correlations for children’s percentages of nonverbal words and their percentages of verbally-based meanings within each grade

Within 3 of the 4 grades, the correlation was slightly above *r* = 0.70 or 50% of common variance (also shown in Table 2).

#### Details of how widely word meanings are known

The levels of nonverbal and verbally-based word meanings known by any children at these ages in Table 3 proved to be almost entirely nonverbal at ages 5 and 6, while some verbally-based meanings were known by some children at ages 7 and 8. Many fewer word meanings were known at ages 5 and 6 than at ages 7 and 8 (based on supplying verbal explanations of meanings).

**Table 3:** Word meaning types by grade and frequency known (sample of 59 word meanings from advantaged classes, Study 2).

Grade and age	Nonverbal meanings known in four grades			
	Most (100%–80%)	Some (79%–40%)	Few (39%–0%)	All words
Pre-kind. (ca 5 years)	0% (0)	27% (8)	73% (22)	100% (30)
Kindergarten (ca 6)	0% (0)	30% (9)	70% (21)	100% (30)
Grade one (ca 7)	20% (6)	43% (13)	37% (11)	100% (30)
Grade two (ca 8)	37% (11)	43% (13)	20% (6)	100% (30)

Word type	Verbally-based meanings known by four grades			
	Most (100%–80%)	Some (79%–40%)	Few (39%–0%)	All words
Pre-kind. (ca 5 years)	0% (0)	0% (0)	100% (29)	100% (29)
Kindergarten (ca 6)	0% (0)	3% (1)	97% (28)	100% (29)
Grade one (ca 7)	0% (0)	14% (4)	86% (25)	100% (29)
Grade two (ca 8)	7% (2)	17% (5)	76% (22)	100% (29)

Word meanings tested and passed are shown for pre-kindergarten (Appendices 2A,B) and grade two (Appendices 3A,B).

## Study 3: word meanings known at kindergarten and grades one, two, four, and five (semi-representative sample)

The main purpose of Study 3 was to contrast word type acquisition at grades two and five. These data are taken from Biemiller and Slonim’s (2001) Studies 1 and 3, working with representative English-speaking children. Also included are percentages of meanings (both meaning types) known by children in kindergarten through grade five. I expected was that there would be substantial growth in verbally-based meanings by grade five. This implies greater student attention to the verbally-based meanings acquired between ages 8 and 11, and more ability to comprehend language with verbally-based meanings.

I also analyzed word acquisition of different word types for children with lower-sized vocabularies vs. larger sized vocabularies (median split).

### Methods: data on word meanings known from kindergarten to grade five

#### Sample

For Study 3, two samples of representative English-speaking children in 3 public schools ranging from assisted housing to upper middle-class neighborhoods in a small Canadian city. All children were Caucasian. Combining these 2001 studies, there were 47 kindergarten, 43 grade one, 51 grade two, 43 grade four, and 44 grade five students. (Grade three was omitted because of Provincial tests at that grade level.) Being limited to English-speaking Caucasian children, this study is only representative of a limited population.

#### Choosing words

117 words—includes the previous 59 words used in Biemiller and Slonim’s Study 2 (which used the same selection method used in their Study 1) and an additional 58 words in their Study 3. Choosing words are as described in the Study 2 above. Two additional words were omitted from analysis because students gave two plausible definitions for the test sentences for these words.

#### Procedure

Same as Study 2.

#### Statistical analysis

Analyses of variance and correlations conducted with *Systat 13* (Systat, 2009).

### Results: word meanings known by children by type from kindergarten to grade five (representative sample)

In Study 3 there were the same significant gains in word meanings at higher grades, and more nonverbal than verbally-based meanings. In this representative population, the number of nonverbal meanings known was again substantially correlated with the number of verbally-based meanings known.

#### Percentage correct

Not surprisingly, more words were known at older grades, and more nonverbal meanings were known than verbally-based meanings (Table 4). ANOVA indicated significant difference between nonverbal vs. verbally-based meanings (*F* = 1,644.8; 1 and 223 df, repeated measure) and Grades (*F* = 118.3; 4 and 223 df). The interaction of Type and Grades was also significant (*F* = 10.2; 4 and 223 df). This interaction reflects the fact that nonverbal meanings Type difference were larger (around 30% from grade two up, while the difference was 23% in kindergarten and grade one).

**Table 4:** Percentages of nonverbal and verbally-based meanings by grade and correlation of types (sample of 117 word meanings from representative classes, Study 3).

Grade	*N*	Type of meanings		
		Nonverbal (62 meanings)	Verbally-based (55 meanings)	Correlation nonverbal × verbally-based
Kindergarten	47	26%	4%	0.73
Grade one	43	30%	7%	0.47
Grade two	51	52%	19%	0.69
Grade four^a^	43	59%	30%	0.70
Grade five	44	68%	37%	0.63
Total	228	47%	19%	0.85

^a^Grade three omitted due to provincial testing.

#### Correlations between nonverbal and verbally-based meanings

As in Study 2, there was a high correlation in Table 4 between the percentages of nonverbal and verbally-based meanings across grades (*r* = 0.85, *N* = 228). Within grades, correlations between nonverbal and verbally-based meanings for 3 of the 5 grades were around half of common variance (Table 4). The other grades were a bit lower. Thus, children who have acquired more nonverbal meanings apparently also tended to acquire more verbally-based meanings.

#### Details of vocabulary at grades 2 and 5

The most important finding is that there was the predicted increase in knowledge of verbally-based meanings between grade two and grade five as shown in Table 5. The percentage of verbally-based meanings known by many or some students rose from 22% in grade two to 47% of 55 verbally-based meanings in grade five.

**Table 5:** Word meaning types by grades two and five and frequency known (sample of 117 word meanings from representative classes, Study 3).

Grade	Nonverbal meanings known in grades two and five					All words
	(Age)	*N*	Most (100%–80%)	Some (79%–40%)	Few (39%–0%)	
Grade two	(ca 8)	51	18% (11)	50% (31)	32% (20)	100% (62)
Grade five	(ca 11)	44	50% (31)	31% (19)	19% (12)	100% (62)

Word type	Verbally-based meanings known in grades two and five					All words
	(Age)	*N*	Most (100%–80%)	Some (79%–40%)	Few (39%–0%)	
Grade two	(ca 8)	51	2% (1)	20% (11)	78% (43)	100% (55)
Grade five	(ca 11)	44	16% (9)	31% (17)	53% (29)	100% (55)

#### Grade two children

Table 5 describes the grade two distribution of the types of words and how well known of the 117 words sampled from LWV grade levels 2 to 12 (Table 5 data and Appendices 4A,B—words and LWV meanings by word frequency and grammatical categories).

In grade two, of the sample of meanings known well (80% or more), all were nonverbal except one (11 of 12). The majority of the meanings known by some (79%–40%) of the grade two students of the total pool of 117 meanings were also nonverbal (31) vs. verbally-based (11). However, among the 63 meanings known by few (less than 40%) were 20 nonverbal and 43 verbally-based meanings.

Appendices 4A,B (*nonverbal* and *verbally-based*) list all 117 words and meanings with LWV levels by grammatical category and frequency for grade two.

By grade two, about 77% of all nonverbal word meanings in our sample of LWV grade level 2–12 meanings were known by most or some children. Among sample words known well continued to be mostly nonverbal (Appendices 4A,B). Perceptual meanings (nouns) known well included objects (*fish*) but also events (*flood*) and settings (*café*). There were few verbs (procedural meanings) known well—only *spread, stab,* and *listen* in our study. There was 1 grade two well-known functional meaning—*near*. The only grade two well-known verbally-based word meaning was *math.*

The relatively few verbally-based word meanings (22% of 55 words) were known by some or most. There some of each grammatical category.

#### Grade five children

Table 5 describes the grade five distribution of the types of known words and how well known are the 117 words sampled from LWV grade levels 2 to 12. Overall, there were 62 nonverbal meanings and 55 verbally-based meanings (Table 5 data and Appendices 5A,B—words and LWV meanings by word frequency and grammatical categories).

In grade five, the majority of the word meanings known by some or most were still nonverbal (50). There were 26 verbally-based meanings known by some or most. There were still 12 nonverbal meanings known by few, and 29 verbally-based meanings.

While the majority of new “well-known” words were nonverbal meanings, about a quarter were verbally-based meanings, including *justice, subtract,* and *secure* (Appendices 5A,B). All but one of these “well-known” words had been known by “some” in grade two. The additions to verbally-based word meanings “known by some” included *period* (a time in history), *nation,* and *former* (the first of two). These had been “known by few” in grade two. However, in grade five there remained more than half of our sample of verbally-based word meanings known by few or none. Almost all of these little-known word meanings were assigned to Dale and O’Roarke’s LWV grade levels 8, 10, or 12.

#### Differences between those with larger vocabularies vs smaller vocabularies for nonverbal and verbally-defined words

For this analysis, I identified children with above-median vocabulary vs. those at/below median vocabulary. Results are shown in Table 6.

**Table 6:** Percentage of word meanings known by at/below median vs. above median and by grade (sample of 117 word meanings, Study 3; SDs in parentheses).

Grade median split	*N*	Nonverbal	Verbally-defined	All
**Kindergarten**				
Low	23	17% (6)	0% (1)	7% (3)
Hi	24	35% (8)	7% (4)	22% (6)
**Grade one**				
Low	23	21% (9)	3% (4)	12% (4)
Hi	21	39% (7)	11% (4)	25% (4)
**Grade two**				
Low	27	42% (13)	13% (6)	27% (9)
Hi	24	64% (8)	26% (8)	45% (6)
**Grade four**				
Low	23	49% (9)	23% (10)	36% (8)
Hi	20	71% (7)	39% (9)	55% (7)
**Grade five**				
Low	22	61% (7)	29% (9)	45% (6)
Hi	22	75% (8)	45% (9)	60% (6)

The main effects of Word Type and Grade were significant. Type: *F* = 2,041.76 (d.f. 1, 219, *p < 0*.001; repeated measure). Grade: *F* = 317.12 (d.f. 4, 219, *p* < 0.001). The main effect of Vocabulary median split was constructed so not a meaningful effect. However, interactions with other variables are relevant.

There are differences between the high vocabulary groups and at/below vocabulary groups in nonverbal vs. verbally-defined words in different grades. 3-way interaction: *F* = 4.01 (d.f. 4, 219). The two-way interactions were also significant.

The practical effect was that the difference in *gains* between grade two vs. grade five the at/below median children vs. above median children is not large. In grade five, the gains from grade two were 19 percent point gains for nonverbal meanings and 16 verbally-defined point gains for at/below-median grade fives vs. 11 nonverbal and 19 point verbally-defined gains for above-median grade fives. In other words, if the low-vocabulary children had learned as many words as the high-vocabulary children by grade two, it is possible that they could have been able to build similar vocabularies between grades three and five.

## Discussion

### To summarize

In Study 1, words “used” by age 3 had mostly nonverbal meanings—perceivable, procedural, modificational, or functional (98.5% of 1,331 words classifiable reported used by at least 2/42 children in Hart and Risley, 1999).

In Study 2, we wanted to look at word meanings acquired from age 5 up to age 8 (between pre-kindergarten to grade two). Before grade one, virtually none of the 29 verbally-based tested words were known. Only *subtract* was known by some children in kindergarten. By the end of first grade, 4 verbally-based word meanings were known by some children, and by grade two, 5 of 29 tested verbally-based word meanings were known by some children including one known by most (*subtract*). There were significant gains across grades, and significantly more nonverbal than verbally-based meanings were known. The number of nonverbal and verbally-based meanings were substantially correlated for students in each grade.

In Study 3, Biemiller and Slonim used a larger sample of word meanings from the *Living Word Vocabulary—*117 word meanings—conducted with representative English-speaking students. In grade two, most or some knew 42 of 62 nonverbal meanings, and 12 of 55 verbally-based *meanings.* By grade five, most or some knew 50 of 62 nonverbal meanings, and 26 of 55 verbally-based meanings—large increases. In short, relatively few verbally-based meanings were acquired by grade two, but many by grade five.

Contrasting children at/below median vocabulary vs. those above median vocabulary showed that size of verbally-defined vocabulary acquired *by* grade two was larger for above-median children. The size of verbally-defined vocabulary gains between grades two and five were similar (16 points for at/below-median vs. 19 points for above-median). By grade five, the at/below median children had acquired similar proportions of vocabulary as the above-median group in grade two.

Studies 1 and 3 probably had under-represented disadvantaged samples. Study 2 had all advantaged children.

Study 3’s sample was reflective of a small city in Ontario, which at the time (1998) had very few non-White families.

### What early-acquired vocabulary is important for later comprehension?

#### From Study 1

To build a larger vocabulary by age 3, I hypothesize that parents and caretakers should probably use more words with nonverbal meanings (illustrated concretely). Introducing modificational words probably requires two or more examples that illustrate differences in the modificational characteristics (e.g., contrasting a *small* ball vs. a *large* ball.). A wider range of functors could be introduced and used. Hart and Risley (1995) reported that vocabulary size “used” by age 3 was predictive of vocabulary size by grade three.

#### From Study 2

In Study 2, I hypothesize that relatively few verbally-based meanings should be addressed by teachers in kindergarten, having found that few verbally-based meanings are learned at age 5. Hadley et al. (2021) also reported difficulty in teaching advanced word meanings to young children. Teaching simple quantitative dimensions like *height* and *weight, numbers* to 100, and functors could be helpful. However, by grade one, more verbally-based meanings could be introduced and used. In addition, it is likely that less advantaged children acquire fewer functors by age 4 or 5. (Study 2 only included advantaged children.) These functors may be necessary to facilitate acquiring other verbally-based word meanings.

#### From Study 3

These verbally-based meanings known by *some* children by the end of grade two may be priority meanings for the primary grades—to be addressed as they It would be helpful to classify occur in texts or instruction during the primary grades from kindergarten to grade two. In the future, it would be helpful to classify all the primary priority word meanings in *Words Worth Teaching* by type (Biemiller, 2010a). This should yield a smaller group of mostly verbally-defined priority words for the primary years.

Findings from the analysis of at/below median-*vs* above-median vocabulary children within grades indicated that the size of *gains* of verbally-defined meanings from grades two to five were similar.

#### Implications of the correlation between nonverbal and verbally-based meanings

The strong correlation between percentages of nonverbal and verbally-based meanings is why the PPVT and other picture-based vocabulary assessments in kindergarten or grade one could predict subsequent vocabulary including verbally-based meanings. Several studies show that a larger PPVT vocabulary in kindergarten is predictive of reading comprehension—especially at grade three and later. Would simply expanding the number of nonverbal meanings both prior to and after age 5 ensure the development of more advanced verbally-based language? Probably, but I recommend concentrating on the words that have been acquired by those with larger vocabularies.

### Fostering verbally-based meanings from grade two on

McKeown (2019) has summarized methods of fostering abstract (verbally-defined) meanings in elementary and middle school. She lists:

1. There are many aspects to know about a word, including features of its meaning, situations in which it is used, associations with other words, and how it behaves syntactically in context.
2. Words are polysemous; their meanings are not static but shift according to context. These shifts may be large or subtle; for example, *accommodate* can mean physically providing room for someone and providing for someone’s need or request, or it can take a more metaphorical sense of being able to understand a new idea that may challenge your perspective.
3. Word knowledge is incremental, gradually developing over multiple encounters.

McKeown (2019) notes that many abstract words are polysemous—have several related meanings. Teacher should use multiple examples from which teachers and students could extract the underlying common meaning. (There are also words with completely unrelated meanings, e.g., *fast* as in “speedy” vs. *fast* in “not eating for a period.” McKeown suggests not teaching these at the same time.)

Some early-acquired *verbally-based* meanings are “basic dimensional meanings” (Case, 1985)—e.g., *height, weight, speed, time, color, size, number, social class,* etc. I hypothesize that instruction can include brief teacher explanations offered during reading texts, teacher answers to children’s questions (also peers’ answers), in-text explanations, and more formal vocabulary instruction. This method proved effective with primary grade children in Biemiller and Boote (2006). In addition to quantitative dimensions, examples of verbally-based word meanings could include words like: *justice, react, secure* (free from fear), and *through.*

Acquiring verbally-defined meanings can occur through teacher explanation or through children’s questions or self-constructed word meanings (Biemiller, 2005). Children’s questions or self-meaning construction requires “word consciousness” (Graves, 2006) or word awareness. (A child cannot address an unknown meaning if they do not attend to unknown words.) In preliminary research, I found that sensitivity to unknown words could be fostered. [This preliminary work is summarized in Biemiller (2010a).]

#### Priority words

In *Words Worth Teaching*, I defined “priority words” for instruction as those known by children with large vocabularies but often not by those with smaller vocabularies at the same age (Biemiller, 2010a). I now think that from grade two on that the verbally-based “priority” word meanings may be more important for instruction than “priority” words with nonverbal meanings. Nonverbal meanings can be learned easily (if objects, pictures or actions are physically present or in texts), while verbally-based meanings probably require more direct instruction.

The finding that low-vocabulary students have similar *gains* between grades two and five, suggests that if the low-vocabulary children before grade two might avoid “low-vocabulary” by grade five. But this depends on whether they could learn as many word meanings (mostly nonverbal) by grade two. If so, it is possible that they could attain grade five vocabulary levels now reached by the high-vocabulary grade fives. This remains to be proven—both that it is possible to increase sufficient vocabulary before grade two, and that if that could be done, that most children could reach grade five with adequate levels for continued academic success.

Both acquiring a larger nonverbal vocabulary by age 5—and continuing exposure to “priority” nonverbal vocabulary after age 5 are necessary, in addition to fostering verbally-based meanings from kindergarten on.

#### Re word priorities

Hadley and Mendez (2021) wrote:

Researchers prioritized unknown words and words described as Tier 2 when selecting words. The most frequently selected word type was concrete nouns; more than half of all words selected were concrete in nature (nonverbal meanings—AB). A small percentage of target words appeared on published lists of words recommended for instruction. Word selection varied by context, with different types of words selected for science content vs. narrative/fiction. Finally, there were few relationships between child characteristics and word characteristics.

As Hadley and Mendez comment, these lists of words for instruction focus too much on nonverbal meanings—that are probably learned easily. They need to be introduced and may require brief explanation. However, clarifying or explaining verbally-based meanings will require more instruction. Furthermore, it may be necessary for curriculum developers and teachers to find or construct texts that allow context and attention for needed verbally-based meanings. This should be researched in future curriculum studies.

Lawson-Adams and Dickinson (2020) emphasize teaching word meanings in the elementary years, noting that nonverbal referents (e.g., pictures, short videos, action, and events) may be needed for nonverbal meanings, and possibly also with some verbally-based meanings.

Hadley et al. (2021, p 9) also share the hypothesis that adding nonverbal word meanings is “easier” (imageable) since “abstract” words are very hard to learn for low-vocabulary children at age 6.0 and below. Concentrating on nonverbal meanings for younger students may be a better use of instruction time. Hadley et al. observe that “In other words, the Matthew effect might be due to inadequate explicit instruction, but it may also be attributable to teaching words that are too abstract and disconnected from the conceptual knowledge of beginning language learners.”

I have similar hypotheses about vocabulary interventions with children age 6.0 and younger. The difference is that I hypothesize that these meanings involve direct developmental neurological representation as perceptual, procedural, or functional files (representations). As reported in the present research, acquisition of verbally-based files (representations) only begin to appear at age 6–7 (grade 1) and more at age 7–8 (grade 2).

### Additional observations regarding neurological evidence for word meaning modalities

As noted previously, Tomasello et al. (2017) review neurological research on activation of cortex regions used when word meanings activating procedural areas and perceptual areas in conjunction with auditory words. In addition, the R. Tomasello et al. study reports computer simulations of processing perceptual and procedural words.

The word learning simulations documented the spontaneous emergence of word/symbol-specific, tightly interconnected cell assemblies within the larger networks, each binding articulatory-acoustic wordforms to sensorimotor semantic information. Due to network structure, connectivity, and Hebbian associative learning, which maps neuronal correlations, the emerging ‘semantic circuits’ for object-and action related words (Italics added, AB) exhibited category-specificity primarily in modality preferential areas; the “higher” multimodal connector hub areas central to the network architecture showed only moderate category-specificity.

I look forward to seeing further research on direct neurological evidence on the relationship between both between nonverbal meanings and neurological representations, and between verbally-based meanings and neurological meanings. Verbally-based meanings are not as neurologically localized as the nonverbal meanings per Papagno (2022).

### Limitations

The samples used for these studies are only moderately representative of English-speaking children in N. America. Both Hart and Risley (1999) and Biemiller and Slonim (2001) are not representative of diverse populations in N. America, although both researchers attempted to incorporate children at varying levels of socio-economic status.

I have not attempted to create a more refined dimension of word meaning type, as Uccelli et al. (2014) suggest. Just as the present study has demonstrated developmental changes in word meanings acquired, a more refined system would probably provide additional educationally-relevant developmental trends.

Most of the findings in this research is correlational—observations of associations between age (studied cross-sectionally) and word types acquired. These findings are not *proof* of what should be taught at various ages. Future research should include research on instruction of verbally-defined word meanings, and research on the longitudinal effects of expanding knowledge of nonverbal word meanings for children age 6 and under for their language and literacy in later grades.

## Conclusion

I hypothesize that the type of words to be taught or fostered for children under age 6 should be mostly what I call nonverbal word meanings. The median-split analysis in Study 3 suggests that building stronger nonverbal vocabularies before grade two could be very beneficial. After age 6, and especially by grade two, the word meanings to be taught or fostered would be mainly verbally-based meanings. I expect that some new nonverbal meanings should also be present and addressed in and after grade two but will require less instructional efforts. These predicted beneficial effects of instruction on vocabulary remain to be proven. Note that most of these data were obtained with White children from a range of socio-economic families.

## Data availability statement

The original contributions presented in the study are included in the article/supplementary material, further inquiries can be directed to the corresponding author.

## Ethics statement

I am referring to the studies conducted in the 1980’s and 1990’s. I have recoded vocabulary data from these studies. I assume that the Hart and Risley research was conducted with approval at the University of Kansas. My research with Naomi Slonim was approved by the University of Toronto, Laboratory School at Toronto, and the participating school board and parents in a southern Ontario city. The studies were conducted in accordance with the local legislation and institutional requirements. Written informed consent for participation in this study was provided by the participants’ legal guardians/next of kin.

## Author contributions

AB: Conceptualization, Formal analysis, Investigation, Methodology, Project administration, Validation, Writing – original draft, Writing – review & editing.

## Footnotes

^1^Other terms for nonverbal meanings are “indexical” (per Deacon, 1997; Nelson, 2007), or “concrete” meanings. Verbally-based meanings are also described as “symbolic” per Deacon and Nelson, or “lexical” per Lawson-Adams and Dickinson (2020).

^2^Reading “comprehension” assessment is usually done using standardized tests (e.g., Iowa Test of Basic Skills) with questions about short texts involving both *facts* which mostly involve nonverbal meanings and *inferences* which often include verbally-based meanings.

^3^Note that some wordforms access both perceptual (noun) and procedural (verb) meanings that are related. For example, “John went for a *swim,*” vs. “Let us *swim* across the pond.” This suggests that some meanings share related perceptual (noun-like) and procedural (verb-like) meanings. In speech, which meaning depends on word order, functors, and affix cues.

^4^Note that early-acquired (before age 5) “modificational” meanings are often “binary” (e.g., *tall* vs. *short*), as Brown (1973) suggested. Many other modificational terms learned early are binary, but not all are.

^5^Relational words (e.g., *tall* or *short*) which may be known by age 3.

^6^Or 49%–70%, after adjusting for guessing.
