# Supplementary material for: Word meaning types acquired before vs. after age 5: implications for education
Source: Front Psychol. 2024 Apr 5;15:1280568. doi: 10.3389/fpsyg.2024.1280568 (PMC11027561; doi:10.3389/fpsyg.2024.1280568)
Supplement: Supplementary file 1 [file Data_Sheet_1.docx]

**Appendix 1A.** **Sample of Nonverbal Word Meanings *Used* by Children by Age 3, Sorted by Number of Children Using Word.** (Sample of words from Hart and Risley’s 1361 words used by 2 or more of 42 representational children. I do not have specific meanings for these words. The oral forms used were noted.)

Most LWV level Some LWV level Few LWV level

(100-80%) (79-40%) (39-5%)

***Nouns***

bed 2

water 2

milk 2

eye 2

bug 2

***Verbs***

want 4

put 2

like 2

watch 2

cut 2

***Adjectives***

big 2

***Functors***

a 2

here 2

on 2

this 2

who 4

because 4

if 4

something 4

their 2

already 4

together 4

never 2

also 4

***Nouns***

apple 2

face 2

tree 2

train 2

boat 2

boots 2

ice 2

aunt 2

coffee 4

grass 2

map 2

guy 4

***Verbs***

blow 2

tear 4

taste 4

feed 2

listen 2

***Adjectives***

dirty 2

black 2

clean 2

right 2

***Functors***

about 2

later 2

through 4

***Nouns***

beans 2

moon 2

slipper 2

park 2

inside 2

pancake 2

oven 2

basement 4

spider 2

lap 4

wheel 2

deer 2

sheep 2

cartoon 2

mirror 2

salt 2

hose 2

robot 4

cocoa 4

pretzel 6

tool 4

building 2

bookcase 2

cradle 2

garbage 2

marble 2

sofa 4

tomato 4

ceiling 2

race 2

brick 2

cinnamon 4

feather 2

helicopter 2

nickel 2

pepper 2

saw 2

spot 4

tire 2

washcloth 2

player 2

restaurant 2

***Verbs***

try 2

tie 2

color 2

shop 2

**Appendix 1A, continued**

Most Some Few

(100-80%) (79-40%) (39-5%)

*Verbs (continued)*

comb 2

bother 4

live 2

bet 2

grow 2

spell 4

bark 2

mix 2

trick 4

ride 2

freeze 2

pump 2

swallow 2

hush 2

***Adjectives***

sorry 4

loud 2

tall 2

fat 2

flat 4

chilly 4

awful 2

naked 4

***Functors***

either 4

both 2

somebody 2

as 2

than 4

someone 4

**Appendix 1B.** **Sample of Verbally-based Words *Used* by Children by Age 3, Sorted by Number of Children Using Word.** (Sample of from Hart and Risley’s 1361 words used by 2 or more of 42 representational children. I do not have specific meanings for these words. The oral forms used were noted.)

Most LWV level Some LWV level Few LWV level

***Adjectives***

six 2

***Nouns***

world 6

***Adjectives***

nine 2

sixty 2

**Appendix 2A. 30 Nonverbal Word Meanings and LWV level *Known* by Children by Age 5 (Pre-Kindergarten), Sorted by Number of Children Using Word and Grammatical Category.**

Most Some Few

(100-80%) (79-40%) (39-0%)

*(No word meanings known by most)*

***Nouns***

Flood 2 (an unusual flow of water)

Match 2 (thing to light fire)

Café 2 (eating place)

Throat 2 (passage from stomach to mouth)

Fish 2 (a water animal)

***Verbs***

Done 2 (finished doing—irreg. past tense)

Fuss 4 (cry and scream)

Flown 4 (fly-irreg. past tense)

***Nouns***

Boulder 2 (large rock)

Anchor 4 (big iron hook)

Cobra 6 (kind of snake)

Thigh 6 (upper part of leg)

Stuff 6 (worthless things)

Sliver 8 (ting piece of wood)

Thud 8 (dull sound)

Shimmer 10 (faint gleam)

Tree 12 (rack for shoes or hats)

Cognac 12 (French brandy)

***Verbs***

Buckle 2 (to fasten)

Hope 2 (expect and wish for)

Beat 4 (flap wings)

Stock 6 (to stock and provide)

Peeve 8 (annoy)

Parch 8 (dry up)

Root 10 (to dig around)

Blab 10 (tell secret)

Swoon 12 (to faint)

***Adjectives/Adverbs***

Straight 4 (direct)

Robust 10 (strong and healthy)

***Functor***

Because 4 (for the reason that)

**Appendix 2B. 29 Verbally-based Word Meanings and LWV levels *Known* by Children by Age 5 (Prekindergarten), Sorted by Number of Children Using Word and Grammatical Category.**

Most Some Few

(100-80%) (79-40%) (39-0%)

(No verbally-based word meanings known by most.)

(No verbally-based word meanings known by some.)

***Nouns***

Polo 4 (game played on horses)

Astronomy 4 (about stars)

Victim 6 (injured person)

Junction 6 (joining)

Guard 6 (a defense)

Drama 8 (plays)

Sequence 8 (connect in series)

Vice 10 (evil habit)

Valor 10 (courage)

Alias 10 (false name)

Franchise 10 (chain of businesses)

Question 12 (problem)

Bit 12 item of computer data)

***Verbs***

Subtract 2 (take number from another)

Know 4 (recognise)

Tally 8 (count)

React 10 (act back)

Induct 12 (to bring in)

Dibs 12 (next claim to)

Etch 12 (engrave with acid)

***Adjectives/Adverbs***

Right 4 (exactly)

Secure 6 (free from fear)

Mammoth 6 (huge)

Inquisitive 8 (curious)

Rotary 8 (wheel-like motion)

Vain 10 (conceited)

Destitute 12 (terribly poor)

Popular 12 (representing the people)

***Functor***

Former 10 (the first of two)

**Appendix 3A. 30 Words with Nonverbal Meanings and LWV level *Known* by Children by Age 8 (Grade 2), Sorted by Frequency of Children Defining Word and Grammatical Category.** (LWV meanings tested in parentheses)

Most Some Few

(100 - 80%) (79 - 40%) (39 – 0%)

***Nouns***

Flood 2 (an unusual flow of water)

Café 2 (eating place)

Match 2 (thing to light fire)

Fish 2 (a water animal)

Throat 2 (passage from stomach to mouth)

Anchor 4 (big iron hook)

Cobra 6 (kind of snake)

***Verbs***

Done 2 (finished doing—irreg. past tense)

Hope 2 (expect and wish for)

Buckle 2 (to fasten)

Flown 4 (fly—irreg. past tense)

***Nouns***

Boulder 2 (large rock)

Stuff 6 (worthless things)

Sliver 8 (ting piece of wood)

Thud 8 (dull sound)

***Verbs***

Fuss 4 (cry and scream)

Beat 4 (flap wings)

Stock 6 (to stock and provide)

Peeve 8 (annoy)

Blab 10 (tell secret)

Root 10 (to dig around)

***Adjective/Adverb***

Straight 4 (direct)

***Nouns***

Tree 12 (rack for shoes or hats)

Cognac 12 (French brandy)

***Verbs***

Parch 8 (dry up)

Swoon 12 (to faint)

***Adjective/adverb***

Robust 10 (strong and healthy)

***Functor***

Because 4 (for the reason that)

**Appendix 3B. 29 Verbally-based Meanings and LWV level *Known* by Children by Age 8 (Grade 2), Sorted by Frequency of Children Using Word and Grammatical Category.** (LWV meanings tested in parentheses)

Most Some Few

(100 - 80%) (79 - 40%) (39 – 0%)

***Verbs***

Subtract 2 (take number from another)

***Nouns***

Drama 8 (plays)

***Verbs***

Tally 8 (count)

React 10 (act back)

***Adjective/Adverb***

Right 4 (exactly)

Secure 6 (free from fear)

***Nouns***

Astronomy 4 (about stars)

Polo 4 (game played on horses)

Junction 6 (joining)

Victim 6 (injured person)

Guard 6 (a defense)

Sequence 8 (connect in series)

Valor 10 (courage)

Alias 10 (false name)

Franchise 10 (chain of businesses)

Question 12 (problem)

Bit 12 item of computer data)

***Verbs***

Know 4 (recognise)

Induct 12 (to bring in)

Dibs 12 (next claim to)

Etch 12 (engrave with acid)

***Adjective/Adverb***

Mammoth 6 (huge)

Inquisitive 8 (curious)

Rotary 8 (wheel-like motion)

Vain 10 (conceited)

Destitute 12 (terribly poor)

Popular 12 (representing the people)

***Functor***

Former 10 (the first of two)

**Appendix 4A. Sample of Nonverbal Word Meanings *Known* by Advantaged Children by End of Grade Two, Sorted by Word Type and Known by Most, Some, or Few Children** (LWV meanings tested in parentheses)

**Nonverbal Meanings Known by**

**Most Some Few**

(100-80%) (79-40%) (39-0%)

***Nouns***

throat 2 (passage from stomach to mouth)

flood 2 (an unusual flow of water)

voice 2 (sound from mouth)

fish 2 (a water animal)

match 2 (thing to light fire)

loop 2 (a circled string)

café 2 (eating place)

shot 6 (injection)

***Verbs***

spread 2 (distribute over a surface)

listen 2 (to try to hear)

stab 4 (stick knife into)

***Functors***

near 2 (close)

***Nouns***

shadow 2 (dark spot cast by light)

boulder 2 (large rock)

space 4 (room)

anchor 4 (big iron hook)

possum 4 (animal)

stuff 6 (worthless things)

cobra 6 (snake)

Vaseline 6 (petroleum jelly)

Parcel 6 (package)

sliver 8 (tiny piece of wood)

thud 8 (dull sound)

wad 8 (rolled up bit)

dodo 10 (extinct bird)

Litter 10 (disorder

shimmer 10 (faint gleam)

matting 12 (a straw fabric)

***Verbs***

buckle 2 (to fasten)

drop 2 (fall)

kept 2 (keep—past tense

done 2 (finished doing—irreg.)

hope 2 (to expect and wish for)

flown 4 (fly—past tense)

fuss 4 (cry and scream)

peep 4 (look shyly)

stock 6 (to stock, provide)

lash 6 (to fasten with rope)

peeve 8 (annoy)

root 10 (to dig around)

blab 10 (tell secret)

l

***Adjectives***

fresh 2 (new, not spoiled)

straight 4 (direct)

***Nouns***

gull 6 (bird)

thigh 6 (upper part of leg)

cartilage 8 (tough tissue)

duplex 8 (two homes in one)

knoll 10 (small hill)

tree 12 (rack for shoes)

cognac 12 (French brandy)

***Verbs***

beat 4 (flap wings)

whittle 4 (to cut wood with a knife)

lance 8 (to cut open)

parch 8 (dry up)

envelop 8 (surround)

lust 10 (strong desire)

swoon 12 (faint)e

garble 12 (to mix up)

writhe 12 (to twist about)

***Adjectives***

robust 8 (strong, healthy)

abrasive 12 (scratch material)

***Functors***

because 4 (for the reason that)

**Appendix 4B. Sample of Verbally-based *Meanings* known Meanings Children by End of Grade Two, Sorted by Word Type and Known by Most, Some, or Few Children** (LWV meanings tested in parentheses)

**Word-based Meanings Known by**

Most  Some Few

(100-80%) (79-40%) (39-0%)

***Nouns***

math 6 (school subject)

***Nouns***

justice 4 (fair dealing)

drama *6*  (plays

haul 10 (distance travelled)

***Verbs***

subtract 2 (take number from another)

react 10 (act back)

tally 8 (count)

know 4 (recognize)

*swing* 4 (swing at a ball in baseball)

***Adjectives***

secure 6 (free from fear)

right 4 (exactly)

***Functor***

through 4 (from start to end)

***Nouns***

man 2 (humankind)

astronomy 4 (about stars)

nation 4 (a country)

period 4 (a time in history)

polo 4 (game played on horseback’)

guard 6 (a defense)

delinquent 6 (breaks laws)

victim 6 (injured person)

junction 6 (joining)

text 6 (schoolbook)

cow 6 (a female of the species)

character 8 (nature of)

republic 8 (representative government)

transit 8 (public transportation)

sequence 8 (connect in series)

locomotion 8 (ability to move)

vice 10 (evil habit)

alias 10 (false name)

jurisdiction 10 (authority)

valor 10 (courage)

matron 10 (married woman)

franchise 10 (chain of businesses)

bit 12 (item of computer data)

question 12 (problem)

oligarchy 12 (ruled by a few)

reformation 12 (protestant era)

discord 12 (clash of sounds)

***Verbs***

dibs 12 (next claim to)

induct 12 (to bring in)

etch 12 (engrave with acid)

***Adjectives***

distant 6 (long ago)

mammoth 6 (huge)

perpendicular 8 (upright position)

inquisitive 8 (curious)

rotary 8 (wheel-like motion)

narrow 10 (lacking a broad view)

curious 10 (odd, strange)

vain 10 (conceited)

ominous 12 (threatening)

destitute 12 (terribly poor0

***Functor***

former 10 (the first of two)

**Appendix 5A. Sample of Nonverbal Word Meanings *Known* by Children by End of Grade Five, Sorted by Word Type and Known by Most, Some, or Few Children.** (LWV meanings tested in parentheses.)

**Nonverbal Meanings Known by**

Most Some Few

(100-80%) (79-40%) (39-0)

***Nouns***

throat 2 (passage from stomach to mouth)

flood 2 (an unusual flow of water)

voice 2 (sound from mouth)

café 2 (eating place)

fish 2 (a water animal)

match 2 (thing to light fire)

loop 2 (a circled string)

boulder 2 (large rock)

anchor 4 (big iron hook)

space 4 (room)

stuff 6 (worthless things)

shot 6 (injection)

cobra 6 (snake)

gull 6 (bird)

sliver 8 (tiny piece of wood)

thud 8 (dull sound)

shimmer 10 (faint gleam)

***Verbs***

spread 2 (distribute over a surface)

listen 2 (to try to hear)

drop 2 (fall)

hope 2 (to expect and wish for)

kept 2 (keep—irreg. past tense)

done 2 (finished doing—irreg.)

flown 4 (fly—irreg. past tense)

stab 4 (stick knife into)

peep 4 (look shyly)

stock 6 (to stock, provide)

peeve 8 (annoy)

blab 10 (tell secret)

***Adjectives***

fresh 2 (new, not spoiled)

***Functor***

near 2 (close)

***Nouns***

shadow 2 (dark spot cast by light)

possum 4 (animal)

parcel 6 (package)

Vaseline 6 (petroleum jelly)

thigh 6 (upper part of a leg)

wad 8 (rolled up bit)

dodo 10 (extinct bird)

litter 10 (disorder)

matting 12 (a straw fabric)

matting 12 (a straw fabric)

tree 12 (rack for shoes, hats)

***Verbs***

buckle 2 (to fasten)

fuss 4 (cry and scream)

beat 4 (flap wings)

lash 6 (to fasten with rope)

envelop 8 (surround)

root 10 (to dig around)

garble 12 (to mix up)

***Adjective***

straight 4 (direct)

***Functor***

because 4 (for the reason that)

***Nouns***

duplex 8 (two homes in one)

cartilage 8 (tough tissue)

knoll 10 (small hill)

cognac 12 (French brandy)

***Verbs***

whittle 4 (to cut wood with a knife)

lance 8 (to cut open)

parch 8 (to dry up)

lust 10 (strong desire)

swoon 12 (to faint)

writhe 12 (to twist about)

***Adjectives***

robust 8 (strong and healthy)

abrasive 12 (scratch material)

ominous 12 (threatening)

**Appendix 5B. Sample of Verbally-based Word Meanings Known by Children by End of Grade Five, Sorted by Word Type and Known by Most, Some, or Few Children.** (LWV meanings in parentheses.)

Word-based Meanings Known by

Most (100 - 80%) Some (79 – 40%) Few (39% or fewe

***Nouns***

justice 4 (fair dealing)

math 6 (school subject)

drama 6 (plays)

***Verbs***

subtract 2 (take number from another)

swing 4 (strike at a ball)

tally 8 (count)

react 10 (act back)

***Adjectives***

secure 6 (free from fear)

distant 6 (long ago)

***Nouns***

man 2 (humankind)

astronomy 4 (about stars)

nation 4 (a country)

period 4 (a time in history)

polo 4 (game played on horseback)

guard 6 (a defense)

victim 6 (injured person)

character 8 (nature of)

transit 8 (public transportation)

haul 10 (distance travelled)

***Verbs***

know 4 (recognize)

dibs 12 (next claim to)

***Adjectives***

right 4 (exactly)

mammoth 6 (huge)

narrow 10 (lacking a broad view)

***Functors***

through 4 (from start to end)

former 10 (the first of two)

***Nouns***

text 6 (schoolbook)

junction 6 (joining)

delinquent 6 (breaks laws)

cow 6 (female of the species)

locomotion 8 (ability to move)

sequence 8 (connect in series)

republic 8 (representative government)

vice 10 (evil habit)

alias 10 (false name)

jurisdiction 10 (authority)

franchise 10 (chain of businesses)

valor 10 (courage)

matron 10 (married woman)

bit 12 (item of computer data)

discord 12 (clash of sounds)

question 12 (problem

oligarchy 12 (ruled by a few)

reformation 12 (Protestant era)

***Verbs***

etch 12 (engrave with acid)

induct 12 (to bring in)

***Adjectives***

rotary 8 (wheel-like motion)

inquisitive 8 (curious)

perpendicular 8 (upright position)

curious 10 (odd, strange)

popular 10 (representing the people)

vain 10 (conceited)

empty 10 (without meaning)

ominous 12 (threatening)

destitute 12 (terribly poor)

***Functor***

former 10 (the first of two)

2396 words in appendices
